# *Lectures on Surgical Pathology*, Delivered at the Royal College of Surgeons of England

**Published:** 1854-02

**Authors:** 


ART. VI.—*Lectures on Surgical Pathology*, delivered at the Royal College of Surgeons of England. By JAMES PAGET, F. R. S., &c., &c., &c. Hypertrophy; Atrophy; Repair; Inflammation; Mortification: Specific Diseases; and Tumors. Philadelphia: Lindsay & Blakiston. 1854.

When we received this work from the publishers, we handed it to a surgical friend for review, in the hope that his superior knowledge of the subjects involved would enable us to furnish a better review than any we could ourselves produce. The lack of time incident to busy professional life has disappointed this hope, and thrown the work back upon our less competent hands.

We are, however, unwilling to pass it by with a casual word of commendation. Surgical pathology, as here treated, is a subject of too great importance, and too much interest. In the work before us we have embodied a clear, intelligible *resume* of existing knowledge. In all the different subdivisions enumerated in the title page, the revelations of the microscope are brought to bear, and an attempt has been made—to our sense a successful one—to render the knowledge thus gained, available in a practical way. While, of course, the treatment of surgical disease has no direct mention, the operative surgeon will find in it an aid in deciding as to the propriety and prognosis of operations, aside from the valuable assistance it will render in cases of doubtful diagnosis.

In the consideration of the subject of nutrition, (which lies at the foundation of all knowledge of disease, and its reparative processes,) our author instances the gradual growth, perfection, and decay of the hair, or deciduous teeth, as going to show that each variety of organization has its appointed time of life, varying in length with the different tissues, and with the retarding or hastening influences of disease, but ever pursuing the same course of development from the cell, to perfection, and subsequent degeneration and absorption, or death and casting off.

To the perfection of this process of nutrition the following conditions are necessary:

"1. A right state and composition of the blood, or other nutritive material.

"2. A regular and not far distant supply of such blood.

"3. (At least in most cases) a certain influence of the nervous system.

"4. A natural state of the part to be maintained."

The author uses the term "right state," rather than "purity" of the blood, for the reason that the term "purity" implies a condition of uniformity in the constituents of the blood and their proportions which does not exist, inasmuch as the blood and tissues are adapted to each other, to their necessities, and to the outward circumstances which surround them, so that health depends, not so much upon any definite proportion of blood elements, as upon the maintenance of this adaptation of the parts of the blood to the wants of the tissues, and *vice-versa*. A quotation will illustrate:

"The necessity for this right or appropriate state of the blood, as a condition of healthy nutrition, involves, of course, the necessity for the due performance of the blood-making and blood-purifying functions; it requires healthy digestion, healthy respiration, healthy excretion. Any one of these being disturbed, the formative process in a part or in the whole body may be faulty, for want of the appropriate material. But, important as these are, we must not let the consideration of them lead us to forget that there is something in the blood itself, which is at least as essential to the continuance of its right and healthy state as these are, and which is, indeed, often occupied in correcting the errors to which these, more than itself, are subject: I mean the power of assimilation or maintenance which the blood possesses, in and for itself, as perfectly and at least as independently as any of the tissues. By this it is, that notwithstanding the diversity of materials put into the blood, and the diversity of conditions in which the functions ministering to its formation are discharged, yet the blood throughout life retains, in each person, certain characters as peculiar as those of his outer features, for the continual renewal of which it provides appropriate materials. And by this assimilative power of the blood it is that the tissues are continually guarded; for by it many noxious substances introduced into the blood are changed and made harmless before they come to the tissues; nor can any substance, introduced from without, produce disease in an organ, unless it be such an one as can escape the assimilative and excretory power of the blood itself.

"In this maintenance is the chief manifestation of the life of the adult-blood; a life, in all essential things, parallel and concurrent with that of the issues. For in the blood we may trace all those which we recognize as signs and parts of life in the solids: we watch its development, its growth, its maintenance by the assimilation of things unlike itself; we find it constituting an adapted purposive part of the organism; possessing organic structures; capable of disease and of recovery; prone to degeneration and to death. In all these things, we have to study the life of the blood as we do that of the solid tissues; the life, not only of the structures of the blood, but of its liquid also; and as, in first development, the blood and tissues are made, of similar materials, in exact conformity with one another, so, through later life, the normal changes of each concur to maintain a like conformity and mutual adaptation. I cannot now dwell on these points;* but they will be frequently illustrated in the following lectures, and some of them at once, in what I have to say of the precision of adjustment in which the "right state" of the blood consists.

"Notwithstanding its possession of the capacity of maintenance, the blood is subject to various diseases, in consequence of which the nutrition of one or more tissues is disordered. The researches of modern chemistry have detected some of these changes; finding excesses or deficiencies of some of the chief constituents of the blood, and detecting in it some of the materials introduced from without. But a far greater number of the morbid conditions of the blood consist in changes from the discovery of which the acutest chemistry seems yet far distant, and for the illustration and discussion of which we cannot adopt the facts, though we may adopt the language and the analogies, of chemistry. It is in such diseases as these that we can best discern how nice is that refinement of mutual influence, how exact and constant that adaptation, between the blood and tissues, on which health depends.

"I know no instance so well adapted to illustrate this as the examples of symmetrical diseases. The uniform character of such diseases is, that a certain morbid change of structure on one side of the body is repeated in the exactly corresponding part on the other side. In the lion's pelvis, for example, which is sketched in the annexed diagram from a specimen, (No. 3030,) in the College Museum, multiform as the pattern is in which the new bone, the product of some disease comparable with a human rheumatism, is deposited — a pattern more complex and irregular than the spots upon a map — there is not one spot or line on one side which is not represented, as exactly as it would be in a mirror, on the other. The likeness has more

* They formed the subject of the course of Lectures delivered at the College in 1848, an abstract of part of which is given by Dr. Kirkes in his "Handbook of Physiology," p. 64, ed. 2.

than Daguerreotype exactness, and was observable in numerous pairs of the bones similarly diseased.

"I need not describe many examples of such diseases. Any out-patient's room will furnish abundant instances of exact symmetry in the eruptions of eczema, lepra, and psoriasis; in the deformities of chronic rheumatism, the paralyses from lead; in the eruptions excited by iodide of potassium or copabia. And any large museum will contain examples of equal symmetry in syphilitic ulcerations of the skull; in rheumatic and syphilitic deposits on the tibiæ and other bones; in all the effects of chronic rheumatic arthritis, whether in the bones, the ligaments, or the cartilages; in the fatty and earthy deposits in the coats of arteries.

"Now the evident and applicable truth in all these cases is, that the morbid substance in the blood, be it what it may, acts upon and changes only certain portions of what we might suppose to be all the very same tissue. Such a substance fastens on certain islands on the surfaces of two bones, or of two parts of the skin, and leaves the rest unscathed; and these islands are the exactly corresponding pieces upon opposite sides of the body. The conclusion is unavoidable, that these are the only two pieces that are exactly alike; that there was less affinity between the morbid material and the osseous tissue, or the skin, or the cartilage, close by; else, it also would have been similarly diseased. Manifestly, when two substances display different relations to a third, their composition cannot be identical; so that though we may speak of all bone or of all skin as if it were all alike, yet there are differences of intimate composition; and in all the body the only parts which are exactly like each other, in their mutual relation with the blood, are those which are symmetrically placed upon the opposite sides. No power of artificial chemistry can, indeed, detect the difference; but a morbid material can: it tests out the parts to which it has the greatest affinity, unites with these, and passes by the rest."*

---

* Some of the differences here noticed are not permanent, but may seem to depend on the several parts of a bone, or of the skin, of a limb (for example) being in different stages of development or degeneration. The symmetrical parts of the tissue, being exactly alike, may be simultaneously and equally affected by a disease, while other parts of the same remain unaffected, till, in the course of time, they attain, by development or degeneration, the very same condition as the parts first affected. Then, if the morbid material still exist in the blood, these parts also become diseased: and so in succession may nearly the whole of a tissue. This view agrees very well with the fact that symmetrical diseases often spread, and so prove that a part which, in one week or

Whatever may be the true explanation of the symmetry of disease, it is becoming evident with advancing chemico-physiology, that we are to look for the cause of changes in tissue, not so much to the quantity or character of elements assimilated through the food, as to the influence which those elements may have upon other sources of supply than that implied in the word food. Gaseous absorption plays an important part in nutrition.

The researches of M. Bernard, mentioned in the Periscope of our last Journal, have a bearing on this. Thus the relative salinity of the blood exerts an important influence on the absorption of oxygen, and the combustion of the tissues. With blood rendered highly saline, a more rapid absorption of oxygen occurs, a consequent increased consumption of carbon, and finally, a waste of the adipose tissue. Now this condition of salinity, while it will increase the appetite, and amount of food consumed, will nevertheless decrease the weight of the body. Thus it is not only the amount of food, but the proportions of inorganic elements, and their action upon the gases, that fill the measure of the necessary conditions of blood changes. The constituents of nutrition may be present in large supply, but the presence of other elements, and chemical reactions, may retard, or hasten the development of tissues, as well as their waste, degeneration, or death.

It will be seen from the turn which this inquiry takes, that surgical pathology does not differ in its ultimate causes, from those lesions confided to therapeutic art, and that surgery is in reality the most readily accessible field for the study of all causation. Turning from this more general view of the subject, we come down to those particular actions which are of immediate interest to the surgeon.

Relative to the repair of wounds, Dr. Paget adopts the idea, first promulgated by Macartney, that wounds closing by "immediate union," do not require that preparatory process of inflammation so long considered necessary. This, he asserts, holds true not only in slight wounds, but often in large incisions, union taking place without the intervention of effused lymph.

"One of the first specimens I examined well illustrated the healing that may now ensue. It was taken from a woman thirty-three years old, whose breast and several axillary glands were removed for cancer. Her general health seemed good, and all went on well after the operation. The flaps,

---

month, is not susceptible of the influence of a morbid material, may, in the next, become as susceptible as that which was first affected. This susceptibility, however, may be due not to normal changes, but to the influence which the diseased portion of the tissue exercises on those around it.

which were of course very large, had been carefully laid down, strapped with isinglass plaster, and well tended. They appeared to unite in the ordinary way, and there remained only a narrow space between their retracted edges, in which space granulations arose from the pectoral muscle. Three weeks after the operation, these were making good progress toward cicatrization; but erysipelas and phlebitis ensued, and the patient died in four or five days.

"I cut off the edges of the wound with the subjacent parts, expecting to find the evidences of union by organized lymph, or, possibly, blood. But neither existed; and the state of parts cannot be better described than by saying that scarcely the least indication remained of either the place where the flap of skin was laid on the fascia, or the means by which they were united. It was not possible to distinguish the relation which these parts held to each other from that which naturally exists between subcutaneous fat and the fascia beneath it. There was no unnatural adhesion; but, as the specimen, which is in the museum of St. Bartholomew's, will still show, the subcutaneous fat which did lie over the mammary gland was now connected with the fascia over the pectoral muscle, just as (for example) the corresponding fat below the clavicle is naturally connected to the portion of the same fascia that lies there. The parts were altered in their relations, but not in their structure. I could find small points of induration where, I suspect, ligatures had been tied, or where, possibly, some slight inflammation had been otherwise excited; and one small abscess existed under the lower flap. But with most careful microscopic examination, I could discover no lymph or exudation-corpuscles, and only small quantities of what looked like the debris of such oil-particles or corpuscules of blood as might have been between the cut surfaces when the flaps were laid down. In short, we cannot otherwise or more minutely describe this healing than by the term "immediate union:" it is immediate, at once in respect of the absence of any intermediate substance placed between the wounded surfaces, and in respect of the speed with which it is accomplished."

He suggests that modern surgeons do not often enough employ those means of bringing solutions of continuity in contact (such as padding the parts to exert gentle pressure) which the old surgeons advocated. Some observations on immediate union in even lacerated wounds by firm compression with adhesive straps, and the treatment of old ulcers by compression, lead us to suppose that this suggestion is a valuable one, and could often be followed without those dangers of inflammation from over-heating, which we so much dread.

It is in the department of morbid growth, and deranged or defective nutrition, as manifested in tumor, cancer, etc., that our author has contributed most to current knowledge. There is a chapter on painful subcutaneous tumors of considerable practical interest. Aside from the ordinarily recognized "painful ganglion," or neuroma, a form of the growth is mentioned where the tumor is so small as to be found with difficulty. It is still a subject of discussion as to whether this disease consists in true growth, or in the entanglement of nerve fibres upon an ordinary tumor. Upon some of these it is impossible to trace the nerve, while in other cases it is found expanded over the surface of the enlargement.

It is, to our sense, not improbable that these cases may exist without actual growth, or tumor, but consisting in some abnormal arrangement of the terminal loops. The tumors are found chiefly upon the lower limb, sometimes as large as a filbert, though generally smaller. Their growth is slow, and they have not much disposition to return. They seem too, to be independent of the general health of the nervous system.

Dr. Warren, of Boston, has recently described a series of cases, all of them, we believe, existing in the fingers, where there was a condition resembling tic-doloreux, and which was relieved by excising the nerve leading to the painful part. A similar case has recently fallen under our notice. For some sixteen years a lady had been subject to daily paroxysms of pain about the root of the nail of the right thumb. Its painfulness depended very much upon uterine, digestive and mental conditions. It first appeared after the birth of a child, and was extremely annoying during subsequent pregnancies, and also whenever a dyspeptic condition, or excitement of mind existed. There was no perceptible tumor, except a little induration from a constant habit of biting the part during paroxysms of pain. The sensations were neuralgic, and though pressure would excite them, they were oftener referable to gastric conditions. For the cure of this, we excised the two dorsal nerves of the thumb, with a good prospect of success, if we may judge from the present relief.

We have already extended this notice to an unusual length, but we wish, before closing, to introduce the author's opinions on the characteristic features of cancer. To this subject large space is devoted.

The recent doubts which have been expressed relative to the uniformity of cancer structure, give to the subject a new interest. The caudate cells have been regarded as peculiar to this disease, until recently, when they have been recognized in other tumors. We quote, somewhat at length, a summary view of this question which it seems to us is (when accompanied by the demonstrations preceding) conclusive as to our ability to recognize cancer by its microscopic structure:

"The developed cancer-structures, if we except the few cases in which they are fibrous or osseous, may be generally described as formed of nucleated cells, or of such corpuscles as are rudimental of, or degenerate from, the nucleated cell. Herein, and in the fact that the corpuscles are neither imbedded in formed intercellular substance, nor orderly arranged, lies one of the characters by which cancers are distinguished from other tumors, and from all natural parts. Their chief heterology, in respect of construction, is in this disorderly crowding of their elements; and I believe it is constant, unless when they imitate the plan of some adjacent natural gland-structure.

"We observe, in the large majority of cancers, two primary or foundation-forms of cells, of which the respective types may be found in gland-cells, and in epithelial or epidermal cells. Of the former, we have examples in the ordinary cells of scirrhous and medullary cancers; of the latter, in the ordinary epithelial cancer-cells; and it is, perhaps, very significant of the meaning of cancer, that the forms which its structures are most prone to assume are after the pattern of those belonging to the natural structures, whose office is to separate whatever is refuse or abnormal from the blood.

"I say, the cancer-cells are formed on the types of excretory gland-cells and epidermal cells; yet, without deviating from the general type, they have special characters by which it is seldom difficult to distinguish them. The question is often asked, What are the characters of the true cancer-cell? or, Has the microscope discovered any structure which is decisive of cancer, wherever it is found? The answers may be:

"(1.) Where cells are found alone, or chiefly composing a tumor, we may be certain that the tumor is a cancer: we may, therefore, regard these as especially cancer-cells.

"(2.) When a tumor is composed, chiefly or alone, of corpuscles, such as the nuclei, or any others which we can trace as rudiments or degenerations of the cancer-cells, the diagnosis of cancer is not less certain: structures such as these are found composing none but cancerous tumors.

"But if the question be changed to,—Are there any cancers which are not formed of structures such as these?—the answer must be affirmative: for there are rare tumors which present the whole clinical history of cancers, and which should, therefore, be called by the same name, though they have not these peculiar cancer-structures, or have them in very subordinate quantity. I do not refer, here, to cancers of which all the structures are imperfect, or degenerate, or diseased; but to such as the fibrous cancers, the osteoid, and certain varieties of the medullary.* These all deviate from the assumed specific cancer-structures; and two of these, the fibrous and osteoid approximate to the characters of natural tissues.

"Together with the disorderly construction, and the peculiar cell-forms, we may often observe, as characteristic of cancers, the multiformity of the structures composing their mass. It is not equaled, I think, by any tumors, unless they be the cartilaginous or the mixed glandular and cartilaginous. The variety of forms appears due, in part, to the mingling of the perfect structures with such as are in various stages of development and degeneration; and, in part, to what seems like a disorderly overgrowth and endogenous increase in cells and their contents. All these forms have been already described; but they may be thus enumerated and arranged:

"(1.) The chief of those to be referred to incomplete development are the free nuclei, and abundant undeveloped liquid or other blastema.

"(2.) The chief forms due to the degeneration are the transitions from cancer-cells or nuclei to granule-masses; the withering corpuscles with fatty degeneration found in the material like tubercle in cancers; the calcerous deposits; the abundant granular matter; and the occasionally mingled melanoid cells.

"(3.) Overgrown or abnormally developed corpuscles are seen in the various extensions of cell walls into angles and processes; and in the enlargement of free nuclei and their assumption of the characters of nucleated cells.

"(4.) The endogenous increase in cells is exemplified in all that is described of the blood-cells and laminated corpuscules of the epithelial and colloid cancers."

From the space which we have devoted to this notice, it will be gathered that we attach to the work a corresponding importance. Those who add it to their libraries will find its peculiar feature in the adaptation of physiology to the history of diseased structure, a fact which gives the work a high intrinsic value. Messrs. Lindsay & Blakiston have issued it in a substantial form, with many illustrations.

For sale by Derby, Orton & Mulligan. S. B. H.

---

* Some pathologists would exclude from the name of cancer all these tumors, and all which are not composed of the "specific" cancer-structures; but I feel sure that we shall do right if (when a choice must be made) we choose modes of life, rather than structures, for determining the affinities of morbid products, and for arrnging them under generic names. As of all tumors, so, especially, of cancers, tho true nature is to be apprehended only by studying them as living things.

